# Height as a risk factor in meningioma: a study of 2 million Israeli adolescents

**DOI:** 10.1186/s12885-020-07292-4

**Published:** 2020-08-20

**Authors:** Matan Ben-Zion Berliner, Lior Haim Katz, Estela Derazne, Hagai Levine, Lital Keinan-Boker, Alexandra Benouaich-Amiel, Omer Gal, Andrew A Kanner, Yosef Laviv, Asaf Honig, Tali Siegal, Jacob Mandel, Gilad Twig, Shlomit Yust-Katz

**Affiliations:** 1 Neuro-Oncology Unit, Davidoff Cancer Center, Rabin Medical Center – Beilinson Hospital, Petach Tikva, Israel; 2 Department of Gastroenterology, Hadassah University Hospital – Ein Kerem, Jerusalem, Israel; 3 Sackler Faculty of Medicine, Tel Aviv University, Tel Aviv, Israel; 4 Braun School of Public Health and Community Medicine, Hadassah University Hospital – Ein Kerem, Jerusalem, Israel; 5 Israel Center for Disease Control, Israel Ministry of Health, Ramat Gan, Israel; 6 School of Public Health, University of Haifa, Haifa, Israel; 7 Department of Neurosurgery, Rabin Medical Center – Beilinson Hospital, Petach Tikva, Israel; 8 Medical Corps, Israel Defense Forces,and Department of Military Medicine, Hebrew University of Jerusalem, Faculty of Medicine, Jerusalem, Israel; 9 Institute of Endocrinology and Talpiot Medical Leadership Program,Sheba Medical Center, Tel Hashomer, Israel

**Keywords:** Allergy, Autoimmune disease, Height, Meningioma, Sex

## Abstract

**Background:**

Meningiomas are the most common primary central nervous system tumors. Potential risk factors include obesity, height, history of allergy/atopy, and autoimmune diseases, but findings are conflicting. This study sought to assess the role of the different risk factors in the development of meningioma in adolescents/young adults.

**Methods:**

The cohort included 2,035,915 Jewish men and women who had undergone compulsory physical examination between 1967 and 2011, at age 16 to 19 years, prior to and independent of actual military enlistment. To determine the incidence of meningioma, the military database was matched with the Israel National Cancer Registry. Cox proportional hazard models were used to estimate the hazard ratios for meningioma according to sex, body mass index (BMI), height, and history of allergic or autoimmune disease.

**Results:**

A total of 480 subjects (328 females) were diagnosed with meningioma during a follow-up of 40,304,078 person-years. Median age at diagnosis was 42.1 ± 9.4 years (range 17.4–62.6). On univariate analysis, female sex (*p* < 0.01) and height (p < 0.01) were associated with risk of meningioma. When the data were stratified by sex, height remained a significant factor only in men. Spline analysis of the male subjects showed that a height of 1.62 m was associated with a minimum disease risk and a height of 1.85+ meters, with a significant risk.

**Conclusions:**

This large population study showed that sex and adolescent height in males (> 1.85 m) were associated with an increased risk of meningioma in adulthood.

## Background

Meningiomas are the most common primary central nervous system tumors. They originate from the meninges which are the membranous layers surrounding the brain. Most meningiomas (80–90%) are grade I (benign); 10–15% are grade II (atypical), and 1–3% are grade III (anaplastic) [1]. Benign meningiomas have a female predominance (2:1 or 3:1) which is not found in the more aggressive types [1]. In the USA, meningiomas were found to be more common in blacks than in whites, with a ratio of 1.2:1 [2]. The risk of acquiring a meningioma increases with age. The median age at diagnosis is 65 years [1].

The only established external (non-genetic) risk factor for brain tumors is exposure to ionizing radiation [3]. An Israeli study revealed abnormally high rates of meningioma in patients treated with low-dose radiation to the scalp for tinea capitis during the 1950s [4]. Other potential risk factors include obesity, height, history of allergy/atopy, and history of autoimmune diseases, but the results are conflicting [5–10]. Establishing risk factors for meningioma can help identify individuals who might benefit from risk reduction strategies and possibly early screening methods.

The aim of the present study was to assess potential risk factors for the development of meningioma in adolescence and early adulthood.

## Methods

### Study population

Israeli adolescents undergo a compulsory medical examination at age 17 to assess their fitness for military service (regardless of whether they are drafted or not). Arab and Orthodox Jewish females and males and Druze females are exempted. Together with the physical examination, sociodemographic and psycho-behavioral data are collected, and the medical history is thoroughly reviewed using documents provided by each subject’s primary care physician. At the end of the process, recruits are assigned a Functional Classification Code (FCC) that describes their medical status and occupational medical ranking. The medical data and FCC are stored in the army’s main database which was computerized in 1967 [11].

**Fig. 1:**
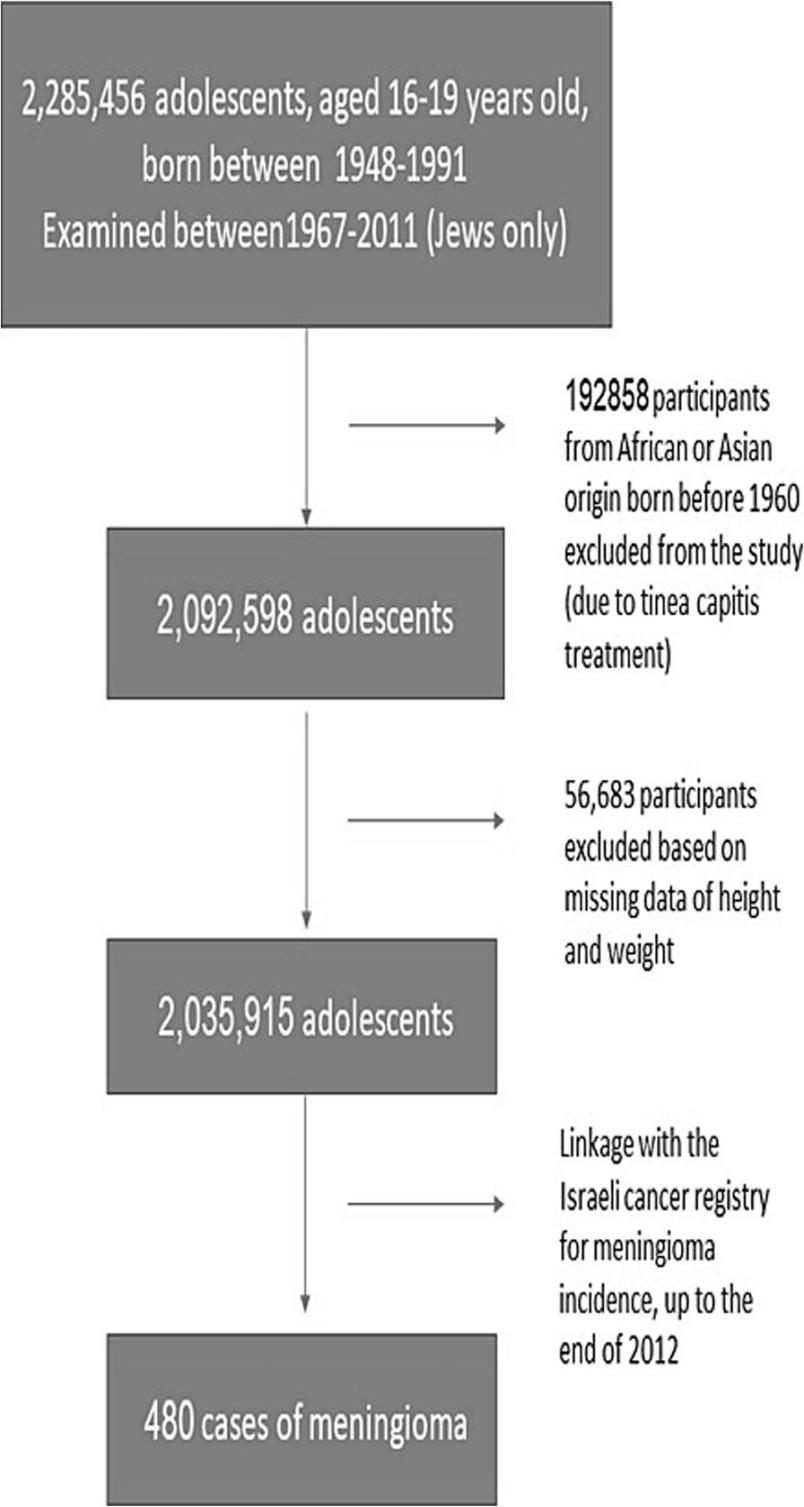
Selection of the study population

The population for the present study was derived from 2,285,456 subjects born in 1948–1991 who underwent pre-enlistment medical examination between 1967, when the database was computerized, and 2011 (at age 16–19 years). Subjects with missing data on height and weight were excluded (*n* = 56,683). We also excluded 192,858 subjects of North African and Asian origin born before 1960, many of whom had been exposed to radiation for the treatment of tinea capitis after immigrating to Israel during the 1950s [12]. These communities were later found to have a particularly high rate of meningioma [4]. The final cohort consisted of 2,035,915 subjects (Fig. 1).

Records were reviewed from the date of the initial medical examination for military fitness to the date of a first diagnosis of any cancer, death, or the predetermined end of follow-up (December 31, 2012).

### Study variables

At the pre-enlistment medical examination, demographic variables for each recruit were recorded in the army database as follows: date of birth, age at examination, country of origin, education, socioeconomic status, height, weight, and body mass index (BMI). Origin was defined by father’s country of birth or, if the examinee’s father was born in Israel, by paternal grandfather’s country of birth, and categorized as Europe (including North and South America, Australia, and Southern Africa), Asia (predominantly the Middle East), Africa (overwhelmingly North Africa), and Israel (third or later generation). Education was categorized as ≤9, 10, 11, and ≥ 12 years of schooling. Socioeconomic status was determined by place of residence at the time of examination, coded on a scale of 1–10, and categorized into low (score 1–4), middle (score 5–7), and high (score 8–10) [13]. Height and weight were measured by trained medics using a stadiometer and a beam balance, with examinees barefoot and in underwear. BMI was calculated as weight (in kilograms) divided by height squared (in meters) and categorized according to the WHO as underweight (< 18.5 kg/m^2^), healthy weight (18.5–24.9 kg/m^2^), overweight (25.0–29.9 kg/m^2^), and obese (≥30.0 kg/m^2^). Height was categorized according to the Centers for Disease Control and Prevention: below 25th percentile, 25th to 50th percentile, 50th to 75th percentile, and 75th percentile and above.

Cognitive function, including language skills and intellectual performance [11], was assessed by a general intelligence test administered by trained personnel. The test is scored on a 90-point scale that is adjusted from time to time; scores are categorized as low (10–30), medium (40–70), and high (80–90) [14].

Medical history was assessed according to the FCCs. Autoimmune diseases (diabetes mellitus, lupus, vasculitis, inflammatory bowel disease, pemphigus, thyroid disease, celiac, rheumatoid arthritis, Addison disease and idiopathic thrombocytopenia purpura) and allergic diseases (asthma, urticaria, eczema, allergic rhinitis, atopic dermatitis, allergic conjunctivitis, and anaphylaxis) were grouped together for the present analysis.

### Ascertainment of meningioma incidence

To determine the incidence of meningioma, we matched the subjects who underwent the pre-enlistment medical examination during the study years, to the Israel National Cancer Registry (INCR), a national, population-based registry established in 1960. In 1982, Israeli law mandated the reportage of all diagnoses of malignant (in situ and invasive), borderline and certain benign (brain and central nervous system) tumors. The estimated rate of reportage for solid tumors is 97%, which meets the standards of the International Association of Cancer Registries (https://www.health.gov.il/PublicationsFiles/ICDC_365_EN_summary.pd). The INCR data include the date of diagnosis, site affected, the International Classification of Diseases code, and the histologic description of the tumor according to the third edition of the International Classification of Diseases for Oncology (ICD-O-3 codes 9350, 95,301, 95,303, 95,310, 95,320, 95,330, 95,340, 95,370, 95,383, and 95,391). At the time of matching, the INCR had been updated until the end of 2012.

### Statistical analysis

Categorical variables are presented as number and percentage, and continuous variables, as mean and standard deviation (SD); median 25th and 75th percentiles, minimum, and maximum were also calculated. The association between risk factors and time to meningioma diagnosis was assessed using Cox proportional hazard models; hazard ratios (HR), 95% confidence intervals (95% CI), and *p*-values were calculated. Log minus log figures were inspected to confirm the proportionality of the hazard. Crude rates were also determined. Independent variables were initially entered individually into the Cox model. After sex and the interaction of sex and height were found to be statistically significant, separate models were established for men and women. A Cox regression cubic spline function with three equally spaced knots positioned between the minimum and maximum values of height was fit to the data to estimate the height value associated with minimum risk of meningioma in men (SAS/STAT and SAS/GRAPH software, version 9.4, SAS Institute Inc., Cary, NC, USA). Other statistical analyses were performed with SPSS Statistics for Windows, version 24.0 (IBM, Armonk, NY, USA). Two-sided *p*-values of ≤0.05 were considered statistically significant.

## Results

### Study population

The baseline characteristics of the study population are presented in Table 1. The mean age at initial examination was 17.4 ± 0.4 years; 41.7% of the cohort was female. The mean duration of follow-up was 19.8 ± 10.5 years (median 18.5) which represent in this study population a follow up of 40,304,078 person-years. The characteristics of the medical history of the subjects are presented in the supplementary table (Table 1S).

**Table 1:** Baseline characteristics of the study population, total and by sex

Characteristics		Male		Female		Total	
		Number	%	Number	%	Number	%
**Birth year**	Total	1,187,421	100.0	848,494	100.0	2,035,915	100.0
	1948–59	117,428	9.9	69,703	8.2	187,131	9.2
	1960–69	256,934	21.6	166,744	19.7	423,678	20.8
	1970–79	358,090	30.2	260,927	30.8	619,017	30.4
	1980–91	454,969	38.3	351,120	41.4	806,089	39.6
**Socioeconomic status**	Low	312,671	26.5	174,640	20.7	487,311	24.1
	Medium	609,644	51.7	456,821	54.1	1,066,465	52.7
	High	257,927	21.9	212,744	25.2	470,671	23.2
**Education**	< 9 years	91,259	7.7	13,631	1.6	104,890	5.2
	10 years	77,372	6.5	24,101	2.8	101,473	5.0
	11 years	98,694	8.3	32,726	3.9	131,420	6.5
	12+ years	919,004	77.5	777,786	91.7	1,696,790	83.4
**Cognitive index**^a^	10–30	193,358	16.4	94,431	11.2	287,789	14.2
	40–70	794,680	67.5	625,924	74.1	1,420,604	70.2
	80–90	189,750	16.1	124,286	14.7	314,036	15.5
**BMI category (Kg/m**^**2**^)	18.5	161,774	13.6	118,713	14.0	280,487	13.8
	18.5 - < 25	866,412	73.0	615,691	72.6	1,482,103	72.8
	25 - < 30	125,021	10.5	90,874	10.7	215,895	10.6
	> 30	33,942	2.9	22,947	2.7	56,889	2.8
**Height category (CDC) (percentile)**	25th percentile	387,793	32.7	226,199	26.7	613,992	30.2
	25th–50th percentile	329,644	27.8	238,839	28.1	568,483	27.9
	50th–75th percentile	285,662	24.1	218,594	25.8	504,256	24.8
	> 75th percentile	184,322	15.5	164,862	19.4	349,184	17.2
**Country of birth**	Europe	155,845	13.1	100,030	11.8	255,875	12.6
	Asia	25,455	2.1	13,374	1.6	38,829	1.9
	Africa	23,734	2.0	10,887	1.3	34,621	1.7
	Israel	981,623	82.7	724,042	85.3	1,705,665	83.8
**Origin**	Europe	542,259	46.1	401,117	47.6	943,376	46.7
	Asia	283,819	24.1	199,373	23.7	483,192	23.9
	Africa	279,824	23.8	190,046	22.5	469,870	23.3
	Israel	69,762	5.9	52,322	6.2	122,084	6.0
		Mean	SD	Mean	SD	Mean	SD
**Age at time of medical examination (years)**		17.4	.5	17.3	.4	17.4	.4
**BMI**		22	3.4	22	3.4	22	3.4
**Height (meters)**		174.0	6.8	162.3	6.1	169.1	8.7

^a^Rated on a 90-point scale

Linkage of the military database with the INCR yielded a diagnosis of meningioma in 480 of the 2,035,915 subjects who underwent medical examination in 1967 to 2011, at age 16–19 years: 228 grade I, 27 atypical, 5 anaplastic, 219 not specified and one patient with meningiomatosis (Table 2). The mean age at diagnosis of meningioma was 42.1 ± 9.4 years (range 17.4–62.6), and at the end of follow-up, 37.2 ± 10.6 years (median 35.9).

**Table 2:** Meningioma type and rate, total and by sex

Meningioma type	Males		Females		Total	
	Number	Per 100,000	Number	Per 100,000	Number	Per 1000,000
**Meningioma NOS**	71	0.3	148	0.9	219	0.54
**Meningiomatosis**	0	0	1	0.006	1	0.002
**Grade 1**	62	0.26	166	1.01	228	0.56
**Atypical + Anaplastic**	19	0.08	13	0.08	32	0.08
**Total**	152	0.63	328	2.00	480	1.19
**Person-years**	23,931,432		16,372,646		40,304,078	

*NOS* Not otherwise specified

### Univariate analysis

**Fig. 2:**
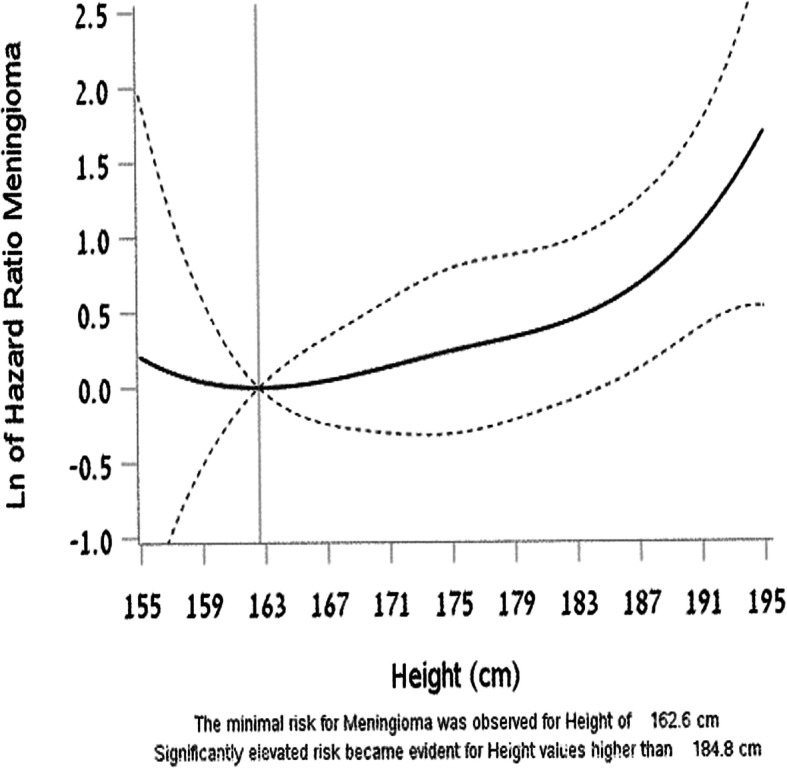
Spline analysis in the men group showing the minimum risk for meningioma at a height of 1.62 m and a statistically significant increase in the risk for meningioma at heights taller than 1.85 m

Overall, as expected, meningiomas were more common in females (328 cases, crude rate 2.0 per 100,000 person years) than in males (152 cases, crude rate 0.63 per 100,000 person-years (*p* < 0.001, HR 3.43 CI 2.84–4.17). However, there was no sex difference in the incidence for the more aggressive meningiomas, atypical and anaplastic (crude rate = 0.08 per 100,000 person-years for males and females). On univariate analysis, only sex and height were significantly associated with the risk of meningioma in the whole study population (*p* < 0.01 for both variables). After stratification by sex, height remained significant only in males (Table 3). The risk of meningioma was minimal when height was up to 1.62 m and statistically significant when height was greater than 1.85 m (Fig. 2). BMI was not associated with an elevated risk of meningioma even when analyzed separately by sex (Table 3).

**Table 3:** Univariate analysis: association of potential risk factors with diagnosis of meningioma, by sex

Variables		Males							Females						
		N	Cases	Crude rate	HR	95% CI Lower Upper		p	N	Cases	Crude rate	HR	95% CI Lower Upper		p
Height	Percentile							0.03							0.12
	< 25%	387,793	46	0.58	1.00				226,199	99	2.32	1.00			
	25–50%	329,644	34	0.55	0.91	0.59	1.42	0.69	238,839	91	1.90	0.77	0.58	1.02	0.07
	50–75%	285,662	40	0.69	1.24	0.81	1.89	0.32	218,594	69	1.66	0.73	0.54	1.00	0.05
	> 75%	184,322	32	0.91	1.76	1.12	2.77	0.01	164,862	69	2.18	0.95	0.70	1.29	0.74
Height continuous					1.03	1.01	1.06	0.02				0.99	0.97	1.01	0.19
BMI	Kg/m^2^							0.43							0.42
	18.5	161,774	16	0.50	0.83	0.49	1.40	0.49	118,713	35	1.60	0.86	0.60	1.22	0.40
	18.5 - < 25	866,412	121	0.68	1.00				615,961	256	2.11	1.00			
	25 - < 30	125,021	10	0.43	0.65	0.34	1.24	0.19	90,874	29	1.73	0.90	0.61	1.32	0.59
	> 30	33,942	4	0.75	1.46	0.54	3.96	0.46	22,947	8	2.25	1.60	0.79	3.23	0.19
Autoimmune diseases^a^	No	1,180,535	152	0.64					841,362	327	2.01	1.00			
	Yes	6886	0	0.00					7132	1	1.07	0.90	0.13	6.44	0.92
Allergic diseases^b^	No	1,050,545	144	0.66	1.00				772,629	307	2.02	1.00			
	Yes	136,876	8	0.36	0.78	0.38	1.60	0.50	75,865	21	1.75	1.29	0.83	2.01	0.26
Asthma	No	1,099,711	148	0.66	1.00				803,877	314	2.01	1.00			
	Yes	87,710	4	0.27	0.59	0.22	1.59	0.29	44,617	14	1.92	1.42	0.83	2.43	0.20
Diabetes	No	1,186,027	152	0.64					847,707	328	2.00				
	Yes	1394	0	0.00					787	0	0.00				

^a^Autoimmune disease: diabetes mellitus, lupus, vasculitis, IBD, pemphigus, thyroid disease, celiac, rheumatoid arthritis, Addison disease and idiopathic thrombocytopenic purpura

^b^Allergic disease including asthma, urticaria, eczema, allergic rhinitis, atopic dermatitis, allergic conjunctivitis, and anaphylaxis

Past medical history of asthma, diabetes, and other atopic or autoimmune diseases was not associated with risk of meningioma. Even when autoimmune and allergic diseases were analyzed as a group, there was no association with lower risk of meningioma (Table 3 and Supplemental Table 2).

When the subjects of African and Asian origin who were excluded from the main analysis were included in the cohort, there was a significant interaction between period of birth (1948–1959 vs 1960–1971) and Asian and African origin (representing the Middle East and North Africa) as opposed to European and Israeli origin. The conjoined effect of birth year and origin showed that origin (North Africa and Asia) was significant only for subjects born between 1948 and 1960 (Supplemental Table 3).

## Discussion

In this nationwide population-based study, we analyzed the association of the development of meningioma in subjects born between 1948 and 1991, with baseline variables obtained for the subjects at the average age of 17 years. As expected, meningiomas were found to be associated with sex (female) and taller stature. None of the other sociodemographic and medical variables assessed, including BMI and a diagnosis of asthma or diabetes at age 17 years, was associated with an increased risk of meningioma.

It is well accepted that benign meningioma is more common in females than males, but the sex predilection disappears with the more aggressive meningiomas [2]. The female predominance might be explained by the finding that meningiomas harbor receptors for estrogen and progesterone [15].

We discovered an association between the risk of meningioma and height in men but not with BMI in men or women. The results of previous studies for these two factors were conflicting. A large Norwegian study including 1.8 million subjects found that height was associated with meningioma in both men and women but BMI was not [5], whereas another study of postmenopausal females revealed an association of meningioma with both BMI and height [6]. A meta-analysis of 12 studies supported the correlation of BMI and meningioma. It is worth noting that the Norwegian study exceeds the meta-analysis in size and power and that in the Norwegian study a subgroup analyses for women and men, as well as different age groups, was performed without finding convincing evidence of a strong association between overweight, obesity and risk for meningioma [5]. In our study, BMI was measured when the subjects were 17 years old, much younger than the studies included in the metanalysis, which might explain the discordant results [8].

Height has been associated with different types of cancer (melanoma, thyroid, testis, breast, and lymphoma). Suggested mechanism for the greater risk of meningioma in taller people is their higher levels of circulating insulin-like growth factors (IGFs), which may influence cell proliferation and tumor growth [16]. Moreover, overexpression of IGF-I and IGF-II mRNA transcripts has been demonstrated in meningioma [17]. Circulating levels of IGFs are highest during puberty. They decrease rapidly in the third decade of life in the general population but seem to stay consistently higher in taller adults [18]. It is not clear why this association was evident only in males in our study, maybe in women the influence of the hormonal status blurred the influence of the height.

Several earlier studies reported an inverse association between a history of allergic diseases (including asthma) and meningioma [9, 10, 18]. However, this finding was not supported by others [19, 20]. We failed to demonstrate an association between meningiomas and allergic diseases including asthma, urticaria, eczema, allergic rhinitis, atopic dermatitis, conjunctivitis, and anaphylaxis and allergy to bees.

Similarly, a recent study reported an inverse association between hyperglycemia and the risk of meningioma [21], whereas another found a positive association with a history of diabetes mellitus [19]. In the present study, diabetes was not associated with the risk of meningioma. This was true for other autoimmune diseases as well.

This analysis also has certain limitations. The follow-up period in this study was limited to 30 years such that the study population was still young when the study ended. Subsequently, the median age of those who developed meningioma in our study was younger than the median age of patients with meningioma in the general population [1]. With a more extensive follow-up, we might find more latent tumor growths that could potentially increase or shift the incidence of intracranial neoplasms. Another limitation of the study is underreporting of meningiomas that are diagnosed only according to radiographic findings (without histological findings). As it is well known that in some cases meningiomas diagnosed radiographically may just be followed by repeat scanning.

Strengths of our cohort include its prospective population-based design, large sample size, high degree of completeness of the cancer registry data throughout the study period, and the ability to carefully control for potential confounders such as exposure to radiation. It should be noted that in a study that was published recently and examined the same cohort, the median height remained almost stable during the study period (the median height of males increased by 3.1 cm and that of females remained stable) despite environmental, social and nutritional changes [22].

## Conclusion

This large population-based study showed that sex (female) and tall stature in adolescent males was associated with an increased risk of meningioma in adulthood.

## Supplementary information

**Additional file 1: Supplementary Table 1**. Medical history characteristics of the study population.

**Additional file 2: Supplementary Table 2**. Univariate analysis: association of potential risk factors with diagnosis of meningioma, by sex.

**Additional file 3: Supplementary Table 3**. Interaction between birth period and origin, whole population adjusted for sex.

## Data Availability

The datasets used during this study are available from the corresponding author on reasonable request.

## Abbreviations

CNS: Central nervous system
BMI: Body mass index
FCC: Functional Classification
INCR: Israel National Cancer Registry
ICD-O: International Classification of Diseases for Oncology
SD: Standard deviation
HR: Hazard ratio
CI: Confidence interval
IGF: Insulin-like growth factor

## References

[1] Ostrom QT, Gittleman H, Fulop J, Liu M, Blanda R, Kromer C, Wolinsky Y, Kruchko C, Barnholtz-Sloan JS (2015). CBTRUS statistical report: primary brain and central nervous system tumors diagnosed in the United States in 2008-2012. Neuro-Oncology.

[2] Claus EB, Bondy ML, Schildkraut JM, Wiemels JL, Wrensch M, Black PM (2005). Epidemiology of intracranial meningioma. Neurosurgery.

[3] Braganza MZ, Kitahara CM, Berrington de González A, Inskip PD, Johnson KJ, Rajaraman P (2012). Ionizing radiation and the risk of brain and central nervous system tumors: a systematic review. Neuro-Oncology.

[4] Modan B, Baidatz D, Mart H, Steinitz R, Levin SG (1974). Radiation-induced head and neck tumours. Lancet.

[5] Wiedmann MKH, Brunborg C, Di Ieva A, Lindemann K, Johannesen TB, Vatten L, Helseth E, Zwart JA (2017). Overweight, obesity and height as risk factors for meningioma, glioma, pituitary adenoma and nerve sheath tumor: a large population-based prospective cohort study. Acta Oncol.

[6] Johnson DR, Olson JE, Vierkant RA, Hammack JE, Wang AH, Folsom AR, Virnig BA, Cerhan JR (2011). Risk factors for meningioma in postmenopausal women: results from the Iowa Women's health study. Neuro-Oncology.

[7] Michaud DS, Bové G, Gallo V, Schlehofer B, Tjønneland A, Olsen A, Overvad K, Dahm CC, Teucher B, Boeing H, Steffen A, Trichopoulou A, Bamia C, Kyrozis A, Sacerdote C, Agnoli C, Palli D, Tumino R, Mattiello A, Bueno-de-Mesquita HB, Peeters PH, May AM, Barricarte A, Chirlaque MD, Dorronsoro M, José Sánchez M, Rodríguez L, Duell EJ, Hallmans G, Melin BS, Manjer J, Borgquist S, Khaw KT, Wareham N, Allen NE, Travis RC, Romieu I, Vineis P, Riboli E (2011). Anthropometric measures, physical activity, and risk of glioma and meningioma in a large prospective cohort study. E Cancer Prev Res (Phila).

[8] Niedermaier T, Behrens G, Schmid D, Schlecht I, Fischer B, Leitzmann MF (2015). Body mass index, physical activity, and risk of adult meningioma and glioma: a meta-analysis. Neurology.

[9] Brenner AV, Linet MS, Fine HA, Shapiro WR, Selker RG, Black PM, Inskip PD (2002). History of allergies and autoimmune diseases and risk of brain tumors in adults. Int J Cancer.

[10] Wang M, Chen C, Qu J, Xu T, Lu Y, Chen J, Wu S (2011). Inverse association between eczema and meningioma: a meta-analysis. Cancer Causes Control.

[11] Gal R (1986). The selection, classification and placement process, in a portrait of the Israeli soldier.

[12] Yust-Katz S, Bar Oz A, Derazne E, Katz LH, Levine H, Keinan-Boker L, Amiel A, Kanner A, Laviv Y, Honig A, Shelef I, Siegal T, Twig G, Kark J (2019). Echoes from the past- changing associations between brain tumors and ethnicity. J Neurol Sci.

[13] Israel Central Bureau of Statistics (2006). Characterization and classification of local authorities by the socio-economic level of the population.

[14] Twig G, Gluzman I, Tirosh A, Gerstein HC, Yaniv G, Afek A, Derazne E, Tzur D, Karasik A, Gordon B, Fruchter E, Lubin G, Rudich A, Cukierman-Yaffe T (2014). Cognitive function and the risk for diabetes among young men. Diabetes Care.

[15] Guevara P, Escobar-Arriaga E, Saavedra-Perez D, Martinez-Rumayor A, Flores-Estrada D, Rembao D, Calderon A, Sotelo J, Arrieta O (2010). Angiogenesis and expression of estrogen and progesterone receptors as predictive factors for recurrence of meningioma. J Neuro-Oncol.

[16] Gunnell D, Oliver SE, Donovan JL, Peters TJ, Gillatt D, Persad R, Hamdy FC, Meal DE, Holly JMP (2004). Do height-related variations in insulin-like growth factors underlie the associations of stature with chronic diseases?. J Clin Endocrinol Metab.

[17] Zumkeller W, Westphal M (2001). The IGF/IGFBP system in CNS malignancy. Mol Pathol.

[18] Crowe FL, Key TJ, Allen NE (2011). A cross-sectional analysis of the associations between adult height, BMI and serum concentrations of IGF-I and IGFBP-1 -2 and −3 in the European prospective investigation into cancer and nutrition (EPIC). Ann Hum Biol.

[19] Berg-Beckhoff G, Schüz J, Blettner M, Münster E, Schlaefer K, Wahrendorf J, Schlehofer B (2009). History of allergic disease and epilepsy and risk of glioma and meningioma (INTERPHONE study group, Germany). Eur J Epidemiol.

[20] Schneider B, Pülhorn H, Röhrig B, Rainov NG (2005). Predisposing conditions and risk factors for development of symptomatic meningioma in adults. Cancer Detect Prev.

[21] Linos E, Raine T, Alonso A, Michaud D (2007). Atopy and risk of brain tumors: a meta-analysis. J Natl Cancer Inst.

[22] Bernardo BM, Orellana RC, Weisband YL, Hammar N, Walldius G, Malmstrom H, Ahlbom A, Feychting M, Schwartzbaum J (2016). Association between prediagnostic glucose, triglycerides, cholesterol and meningioma, and reverse causality. Br J Cancer.

