# Supplementary material for: Height as a risk factor in meningioma: a study of 2 million Israeli adolescents
Source: BMC Cancer. 2020 Aug 20;20:786. doi: 10.1186/s12885-020-07292-4 (PMC7441683; doi:10.1186/s12885-020-07292-4)
Supplement: Supplementary file 1 — Additional file 1: Supplementary Table 1. Medical history characteristics of the study population. [file 12885_2020_7292_MOESM1_ESM.docx]

**Supplementary Table 1 (1S)** Medical history characteristics of the study population

|  |  | Males | | | Females | | |
| --- | --- | --- | --- | --- | --- | --- | --- |
| Disease |  | Total | Meningioma | Crude Rate | Total | Meningioma | Crude Rate |
| Asthma | Yes | 87710 | 4 | 0.27 | 44617 | 14 | 1.92 |
|  | No | 1099711 | 148 | 0.66 | 803877 | 314 | 2.01 |
| IBD | Yes | 897 | 0 | 0 | 448 | 0 | 0 |
|  | No | 1186524 | 152 | 0.64 | 848046 | 328 | 2 |
| Urticaria | Yes | 593 | 0 | 0 | 516 | 0 | 0 |
|  | No | 1186828 | 152 | 0.64 | 847978 | 328 | 2 |
| Diabetes | Yes | 1394 | 0 | 0 | 787 | 0 | 0 |
|  | No | 1186027 | 152 | 0.64 | 847707 | 328 | 2 |
| Thyroid* | Yes | 991 | 0 | 0 | 2731 | 0 | 0 |
|  | No | 118643 | 152 | 0.64 | 854763 | 328 | 2.01 |
| Eczema | Yes | 3528 | 0 | 0 | 2988 | 1 | 2.64 |
|  | No | 1184163 | 152 | 0.64 | 845506 | 327 | 2 |
| Celiac | Yes | 299 | 0 | 0 | 270 | 0 | 0 |
|  | No | 1187122 | 152 | 0.64 | 848224 | 328 | 2 |
| Psoriasis | Yes | 2663 | 0 | 0 | 2200 | 1 | 2.91 |
|  | No | 1184758 | 152 |  | 846294 | 327 | 2 |
| Rheumatoid arthritis | Yes | 119 | 0 | 0 | 175 | 0 | 0 |
|  | No | 1187302 | 152 | 0.64 | 848319 | 328 | 2 |
| Vasculitis | Yes | 62 | 0 | 0 | 137 | 0 | 0 |
|  | No | 1187359 | 152 | 0.64 | 848357 | 328 | 2 |
| Addison | Yes | 15 | 0 | 0 | 15 | 0 | 0 |
|  | No | 1187406 | 152 | 0.64 | 848479 | 328 | 2 |
| Pemphigus | Yes | 7 | 0 | 0 | 3 | 0 | 0 |
|  | No | 1187414 | 152 | 0.64 | 848491 | 328 | 2 |
| Allergic rhinitis | Yes | 66348 | 5 | 0.48 | 37423 | 10 | 1.72 |
|  | No | 1121073 | 147 | 0.64 | 811071 | 318 | 2.01 |
| Anaphylaxis | Yes | 578 | 0 | 0 | 599 | 0 | 0 |
|  | No | 1186843 | 152 | 0.64 | 847859 | 328 | 2 |
| Atopic | Yes | 5575 | 0 | 0 | 5279 | 1 | 1.27 |
|  | No | 1187302 | 152 | 0.64 | 843215 | 327 | 2.01 |
| Atopic dermatitis | Yes | 2453 | 0 | 0 | 2519 | 0 | 0 |
|  | No | 1184968 | 152 | 0.64 | 845975 | 328 | 2.01 |
| Allergic diseases** | Yes | 136876 | 8 | 0.36 | 846145 | 21 | 1.75 |
|  | No | 1050545 | 144 | 0.66 | 772629 | 307 | 2.02 |
| Autoimmune diseases^†^ | Yes | 6886 | 0 | 0 | 7132 | 1 | 1.07 |
|  | No | 1180535 | 152 | 0.64 | 841362 | 327 | 2.01 |

IBD, inflammatory bowel disease.

*Thyroid- Hashimoto thyroiditis, Grave’s disease.

** Allergic disease: asthma, urticaria, eczema, allergic rhinitis, atopic dermatitis, allergic conjunctivitis, and anaphylaxis.

^†^ Autoimmune disease: diabetes mellitus, lupus, vasculitis, IBD, pemphigus, thyroid disease, celiac, rheumatoid arthritis, Addison disease, and idiopathic thrombocytopenia purpura.
