# Supplementary material for: Height as a risk factor in meningioma: a study of 2 million Israeli adolescents
Source: BMC Cancer. 2020 Aug 20;20:786. doi: 10.1186/s12885-020-07292-4 (PMC7441683; doi:10.1186/s12885-020-07292-4)
Supplement: Supplementary file 2 — Additional file 2: Supplementary Table 2. Univariate analysis: association of potential risk factors with diagnosis of meningioma, by sex. [file 12885_2020_7292_MOESM2_ESM.docx]

**Supplementary Table 2 (2S)** Univariate analysis: association of potential risk factors with diagnosis of meningioma, by sex

| **Variables** | **Males** | | | | | | **Females** | | | | | |  |  |  |
| --- | --- | --- | --- | --- | --- | --- | --- | --- | --- | --- | --- | --- | --- | --- | --- |
|  |  | N | Cases | Crude rate | HR | 95% CI | | p | N | Cases | Crude rate | HR | 95% CI | | p |
|  |  |  |  |  |  | Lower | Upper |  |  |  |  |  | Lower | Upper |  |
| **Socioeconomic status** | |  |  |  |  |  |  | 0.62 |  |  |  |  |  |  | 0.32 |
|  | Low | 312671 | 26 | 0.42 | 0.78 | 0.47 | 1.3 | 0.35 | 174640 | 63 | 1.96 | 1.25 | 0.9 | 1.73 | 0.18 |
|  | Medium | 609644 | 90 | 0.74 | 1.29 | 0.87 | 1.91 | 0.2 | 456821 | 176 | 2.03 | 1.19 | 0.92 | 1.53 | 0.19 |
|  | High | 257927 |  | 0.64 | 1 |  |  |  | 212744 | 88 | 2 | 1 |  |  |  |
| **Education** |  |  |  |  |  |  |  | 0.53 |  |  |  |  |  |  | 0.5 |
|  | <9 years | 91259 | 27 | 1.06 | 1.33 | 0.87 | 2.05 | 0.19 | 13631 | 16 | 4.27 | 1.46 | 0.88 | 2.42 | 0.14 |
|  | 10 years | 77372 | 15 | 0.77 | 0.99 | 0.58 | 1.72 | 0.98 | 24101 | 19 | 2.98 | 0.98 | 0.61 | 1.56 | 0.92 |
|  | 11 years | 98694 | 16 | 0.74 | 1.25 | 0.74 | 2.13 | 0.4 | 32726 | 18 | 2.48 | 1.12 | 0.7 | 1.81 | 0.64 |
|  | 12+ years | 919004 | 94 | 0.55 | 1 |  |  |  | 777786 | 275 | 1.88 | 1 |  |  |  |
| **Cognitive index** |  |  |  |  |  |  |  | 0.29 |  |  |  |  |  |  |  |
|  | 10-30 | 193358 | 12 | 0.37 | 1 |  |  |  | 94431 | 16 | 1.2 | 1 |  |  | 0.44 |
|  | 40-70 | 794680 | 111 | 0.69 | 1.34 | 0.74 | 2.45 | 0.33 | 625924 | 239 | 1.99 | 0.86 | 0.51 | 1.43 | 0.56 |
|  | 80-90 | 189750 | 26 | 0.62 | 1 | 0.5 | 2 | 1 | 124286 | 70 | 2.36 | 0.74 | 0.43 | 1.29 | 0.29 |
|  | 18.5 | 161774 | 16 | 0.5 | 0.83 | 0.49 | 1.4 | 0.49 | 118713 | 35 | 1.6 | 0.86 | 0.6 | 1.22 | 0.4 |
|  | 18.5 - <25 | 866412 | 121 | 0.68 | 1 |  |  |  | 615961 | 256 | 2.11 | 1 |  |  |  |
|  | 25 - <30 | 125021 | 10 | 0.43 | 0.65 | 0.34 | 1.24 | 0.19 | 90874 | 29 | 1.73 | 0.9 | 0.61 | 1.32 | 0.59 |
|  | >30 | 33942 | 4 | 0.75 | 1.46 | 0.54 | 3.96 | 0.46 | 22947 | 8 | 2.25 | 1.6 | 0.79 | 3.23 | 0.19 |
| **Origin** |  |  |  |  |  |  |  |  |  |  |  |  |  |  | 0.77 |
|  | Europe | 542259 | 101 | 0.85 | 1 |  |  | 0.35 | 401117 | 195 | 2.32 | 1 |  |  | 0.77 |
|  | Asia | 283819 | 19 | 0.36 | 0.7 | 0.42 | 1.16 | 0.16 | 199373 | 57 | 1.6 | 1.12 | 0.82 | 1.52 | 0.47 |
|  | Africa | 279824 | 22 | 0.43 | 0.8 | 0.5 | 1.29 | 0.36 | 190046 | 54 | 1.63 | 1.16 | 0.85 | 1.59 | 0.35 |
|  | Israel | 69762 | 6 | 0.44 | 0.62 | 0.27 | 1.41 | 0.25 | 52322 | 20 | 2.09 | 1.09 | 0.69 | 1.73 | 0.72 |
| **Country of birth** |  |  |  |  |  |  |  | 0.16 |  |  |  |  |  |  | 0.27 |
|  | Europe | 155845 | 24 | 0.79 | 1.19 | 0.77 | 1.85 | 0.44 | 100030 | 27 | 1.58 | 0.8 | 0.54 | 1.19 | 0.27 |
|  | Asia | 25455 | 4 | 1.04 | 2.62 | 0.96 | 7.11 | 0.06 | 13374 | 1 | 0.54 | 0.44 | 0.06 | 3.15 | 0.41 |
|  | Africa | 23734 | 1 | 0.21 | 0.36 | 0.5 | 2.56 | 0.31 | 10887 | 7 | 3.55 | 1.71 | 0.81 | 3.63 | 0.16 |
|  | Israel | 981623 | 123 | 0.61 | 1 |  |  |  | 724042 | 293 | 2.05 | 1 |  |  |  |
