# Supplementary material for: Height as a risk factor in meningioma: a study of 2 million Israeli adolescents
Source: BMC Cancer. 2020 Aug 20;20:786. doi: 10.1186/s12885-020-07292-4 (PMC7441683; doi:10.1186/s12885-020-07292-4)
Supplement: Supplementary file 3 — Additional file 3: Supplementary Table 3. Interaction between birth period and origin, whole population adjusted for sex. [file 12885_2020_7292_MOESM3_ESM.docx]

**Supplementary Table 3 (3S)** Interaction between birth period and origin, whole population adjusted for sex*

| **Interaction** | **HR** | **95% CI** | | **p value** |
| --- | --- | --- | --- | --- |
|  |  | **Lower** | **Upper** |  |
| **Sex** | 2.75 | 2.38 | 3.18 | <0.001 |
| **Birth period - origin** |  |  |  | <0.001 |
|  |  |  |  |  |
| **Birth period - Origin** |  |  |  |  |
| **1960 or later - Europe + Israel** | 1 |  |  |  |
| **1960 or later - Africa + Asia** | 0.96 | 0.76 | 1.20 | 0.72 |
| **1948-1959 - Europe + Israel** | 0.87 | 0.68 | 1.11 | 0.26 |
| **1948-1959 - Africa + Asia** | 1.58 | 1.26 | 1.98 | <0.001 |
|  |  |  |  |  |

*Birth period was categorized as 1948-1959 vs 1960-1967; origin was categorized as Asia and North Africa vs Europe and Israel.

The analysis included also subjects from North Africa and Asia born before 1960 who were excluded from the main analyses.
